# The small molecule STF-62247 induces apoptotic and autophagic cell death in leukemic cells

**DOI:** 10.18632/oncotarget.25291

**Published:** 2018-06-12

**Authors:** Tomohiro Kozako, Keisuke Sato, Yuichiro Uchida, Naho Kato, Akiyoshi Aikawa, Kentaro Ogata, Hidetoshi Kamimura, Haruna Uemura, Makoto Yoshimitsu, Kenji Ishitsuka, Yasuki Higaki, Hiroaki Tanaka, Shin-Ichiro Honda, Shinji Soeda

**Affiliations:** ^1^ Department of Biochemistry, Faculty of Pharmaceutical Sciences, Fukuoka University, Fukuoka, Japan; ^2^ Department of Pharmacy, Fukuoka University Hospital, Fukuoka, Japan; ^3^ Division of Hematology and Immunology, Center for Chronic Viral Diseases, Graduate School of Medical and Dental Sciences, Kagoshima University, Kagoshima, Japan; ^4^ Department of Clinical Pharmacology, Faculty of Pharmaceutical Sciences, Fukuoka University, Fukuoka, Japan; ^5^ Department of Hematology and Immunology, Kagoshima University Hospital, Kagoshima, Japan; ^6^ Faculty of Sports and Health Science, Fukuoka University, Fukuoka, Japan

**Keywords:** Human T cell leukemia virus-1, adult T cell leukemia/lymphoma, apoptosis, autophagy, STF-62247

## Abstract

Adult T cell leukemia/lymphoma (ATL) is an aggressive malignant T cell disease caused by human T cell leukemia virus-I (HTLV-1). Treatment outcomes for aggressive subtypes of ATL remain poor, with little improvement in overall survival since HTLV-1 was discovered. Therefore, new therapeutic strategies for ATL are required. STF-62247 induces autophagy and selectively kills renal cell carcinoma without apoptotic cell death. Here, we demonstrate that STF-62247 reduced cell viability and resulted in autophagosome accumulation and autophagy in leukemic cell lines (S1T, MT-2, and Jurkat). Interestingly, STF-62247 induced apoptosis in HTLV-1-infected cell lines (S1T and MT-2), as indicated by DNA fragmentation and caspase activation, but not in non-HTLV-1-infected Jurkat cells; a caspase inhibitor did not prevent this caspase-associated cell death. STF-62247 also increased nuclear endonuclease G levels. Furthermore, STF-62247 reduced cell viability and increased the number of apoptotic cells in peripheral blood mononuclear cells collected from patients with acute ATL, which has a poor prognosis. Therefore, STF-62247 may have novel therapeutic potential for ATL. This is the first evidence to demonstrate the cell growth-inhibitory effect of an autophagy inducer by caspase-dependent apoptosis and caspase-independent cell death via autophagy and endonuclease G in leukemic cells.

## INTRODUCTION

Adult T cell leukemia/lymphoma (ATL), a malignancy of peripheral CD4^+^ T cells caused by human T cell leukemia virus-I (HTLV-1), is a retrovirus infecting approximately 10–15 million people worldwide, mainly in southern Japan, the Caribbean basin, South America, Melanesia, and Equatorial Africa [1–3]. There are four subtypes of ATL: acute, lymphoma, chronic, and smoldering [4]. Intensive multiagent chemotherapies with or without subsequent allogeneic hematopoietic stem cell transplantation are usually recommended for acute, lymphoma, or unfavorable chronic subtypes (aggressive ATL) [5, 6]. However, treatment outcomes for aggressive subtypes of ATL remain poor, with little improvement in overall survival in the 35 years since HTLV-1 was discovered. Thus, despite recent progress in treatment modalities [7], new therapeutic strategies for ATL are required.

Autophagy is a lysosome-dependent process by which the cell catabolizes its own cytoplasmic organelles [8]. Autophagy can be activated in response to multiple stresses and has been demonstrated to promote tumor cell survival and drug resistance [9]. Alternatively, it can be tumor suppressive through the elimination of oncogenic protein substrates, damaged organelles, and toxic unfolded proteins [10]. Deregulation of autophagy occurs in multiple types of cancer, with current research supporting a complex and cellular context-specific role for this process during oncogenic transformation. Thus, autophagy appears to be tumor suppressive in normal cells and during early oncogenic transformation, but may act as a critical survival pathway for established tumors [9, 11]. As such, defining context-dependent roles for autophagy in cancer and the mechanisms involved will be important to conduct autophagy-based therapeutic intervention. Recently, the autophagy inducer STF-62247 was identified as selectively toxic and growth inhibitory to renal cell carcinoma lacking Von Hippel-Lindau (VHL) tumor suppressor activity, an effect that occurred without apoptosis [12].

The HTLV-1 Tax protein increases autophagosome accumulation as part of the autophagy pathway, thus playing a protective role during death receptor-mediated apoptosis [13]. Death receptor-mediated apoptosis is correlated with Tax degradation, which can be facilitated by inhibitors of autophagy. Recently, we reported that small-molecule sirtuin 1 inhibitors induce cell death, as indicated by DNA fragmentation and autophagic markers, in HTLV-1-infected cell lines with or without Tax [14]. In contrast, STF-62247 is a cell-permeable autophagy inducer that selectively induces non-apoptotic cell death in VHL-deficient, but not VHL-expressing, renal carcinoma cells (RCC) both *in vitro* and *in vivo*. The extent to which STF-62247 can promote the induction of chemically induced cell death in HTLV-1-infected cells and ATL is currently unclear.

Here, we assessed how STF-62247 affects leukemic cell lines. We found that STF-62247 reduced cell viability by increasing autophagy. Interestingly, STF-62247 also enhanced DNA fragmentation, phosphatidylserine externalization, and caspase activation in HTLV-1-infected cell lines. STF-62247 also increased nuclear endonuclease G levels. Furthermore, STF-62247 reduced cell viability and increased the number of apoptotic cells in peripheral blood mononuclear cells (PBMCs) collected from patients with acute ATL. As STF-62247 induced cell death via apoptosis and caspase-independent autophagic cell death, it may have novel therapeutic potential for treatment of ATL.

## RESULTS

### STF-62247 induced cell death of leukemic cell lines

**Figure 1:**
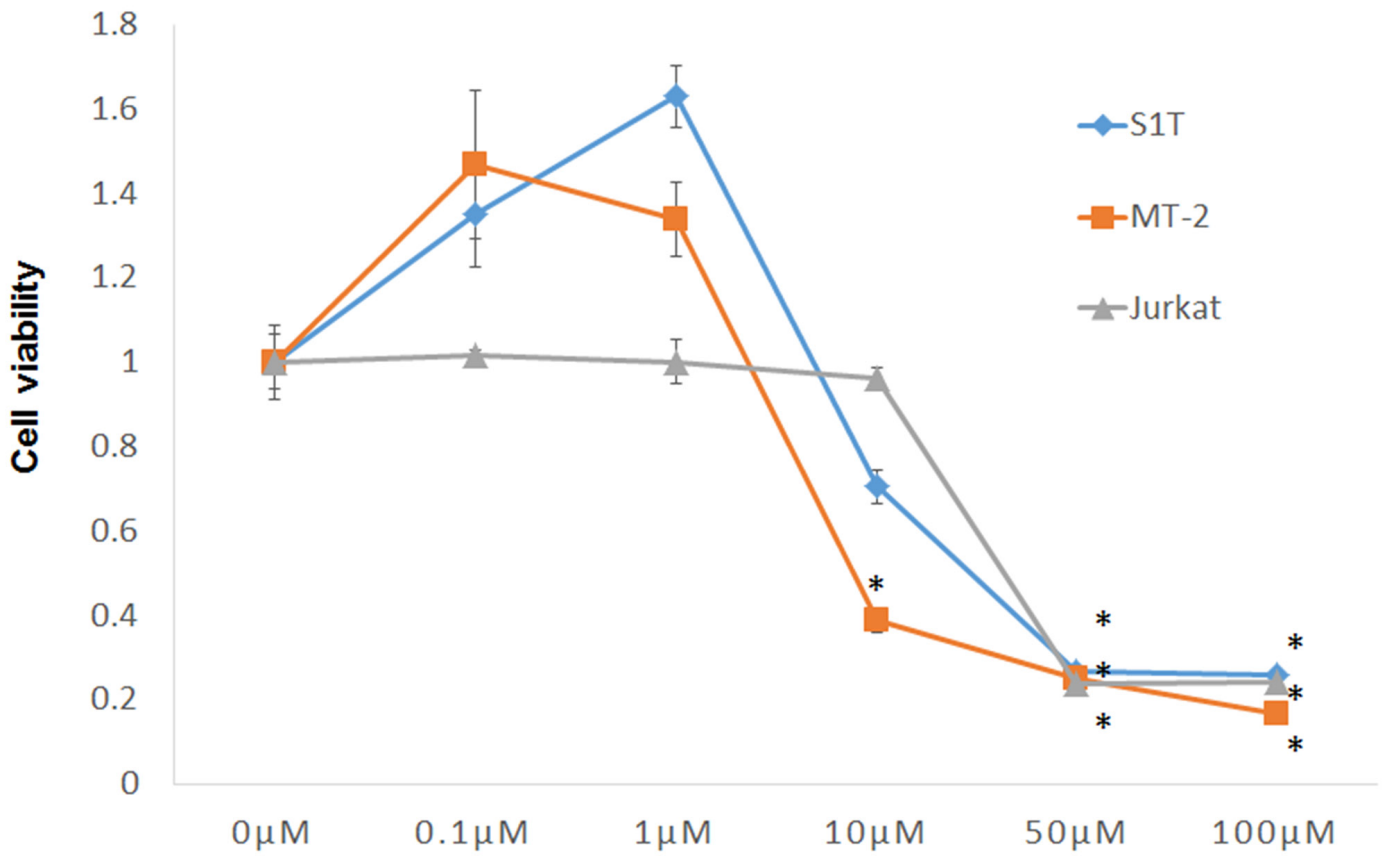
STF-62247 induced cell death of leukemic cell lines Cell lines were incubated at 2 × 10^5^ cells/mL in the presence of indicated concentrations of STF-62247 for 72 h. Data represent mean percentage ± SD of three independent experiments. ^*^P < 0.01 vs. 0 μM STF-62247.

The effect of STF-62247 on viability of S1T, MT-2, and Jurkat cells was examined using Cell Count Reagent SF. STF-62247 inhibited growth of all three cell lines in a dose-dependent manner (Figure 1). Indeed, STF-62247 demonstrated potent activity (96 h) with GI_50_ values of 14.9, 6.4, and 19.3 μM for S1T, MT-2, and Jurkat cells, respectively (Table 1). Thus, MT-2 cells were most sensitive to STF-62247-induced cell death.

**Table 1:** GI_50_ of STF-62247 in leukemic cell lines

Cell line	24 h	48 h	72 h	96 h
S1T	43.0	29.3	21.2	14.9
MT-2	42.9	15.1	7.62	6.43
Jurkat	83.9	37.6	27.9	19.3

Cell lines were incubated at 2 × 10^5^ cells/mL in the presence of indicated concentrations (μM) of STF-62247 for 24–96 h. Values of GI_50_ (compound concentration that reduced cell growth by 50%) are shown for various time points.

### STF-62247 induced autophagy in leukemic cell lines

**Figure 2:**
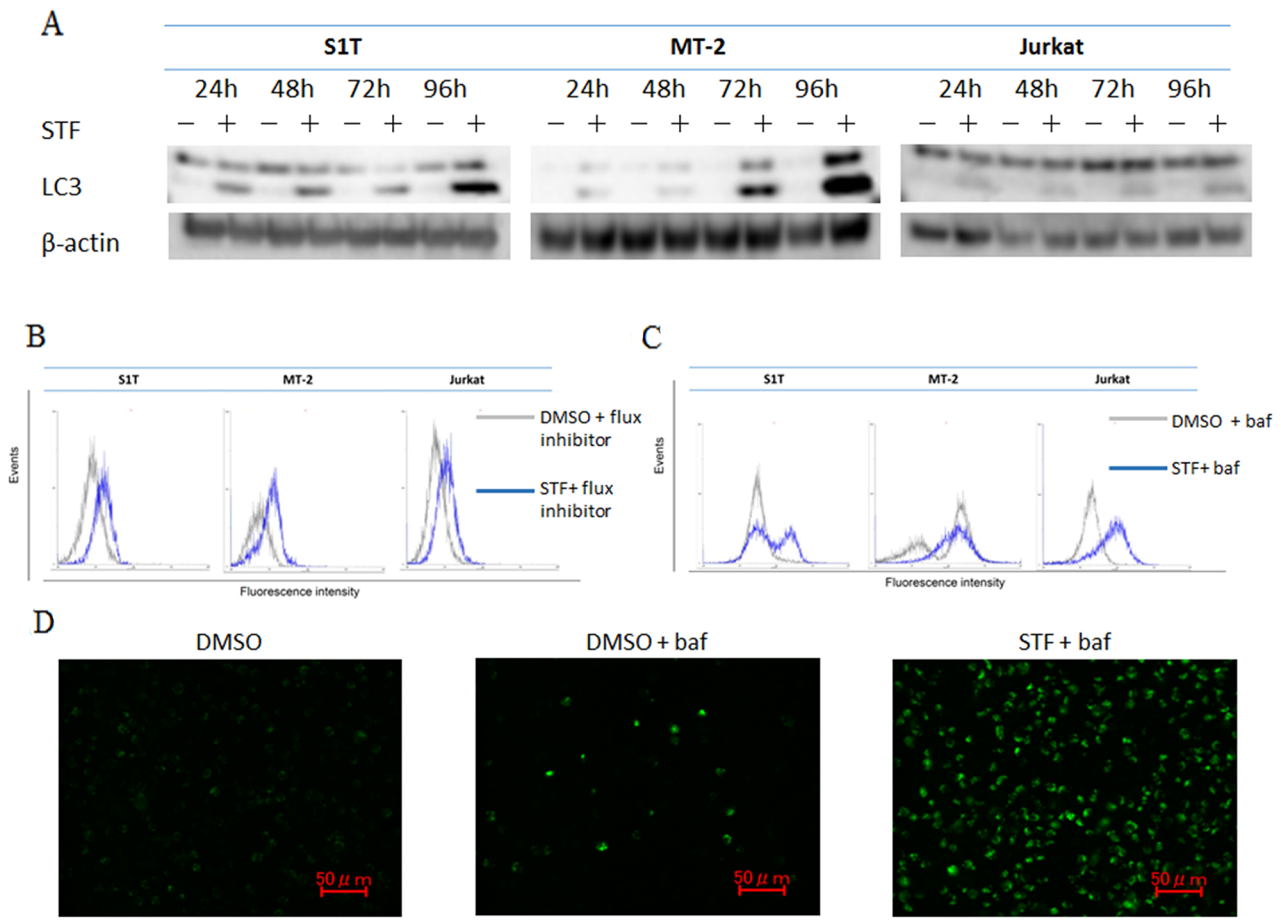
STF-62247 induced autophagy S1T, MT-2, and Jurkat cells were treated with STF-62247 (STF; S1T and Jurkat: 50 μM; MT-2: 10 μM). **(A)** Protein levels were detected by western blotting with indicated antibodies. **(B)** Autophagy was measured by quantifying mean fluorescence intensity of LC3-II using FlowCollect™. Cells were pre-incubated for 30 min with the autophagy flux inhibitor provided prior to treatment with LC3 antibody. **(C, D)** Cellular autophagic flux after 48-h STF-62247 treatment was evaluated using flow cytometry and fluorescence microscopy. Representative fluorescence microscopy data for S1T are indicated. Cells were pre-incubated for 30 min with bafilomycin A1 (baf) prior to treatment of CYTO-ID® Green Detection Reagent.

Multiple studies have shown that genetic knockdown of autophagy-related Atg proteins or pharmacological inhibition of autophagy can effectively enhance tumor cell death induced by a diverse array of anticancer drugs in preclinical models [15]. Paradoxically, autophagy defects are associated with increased tumorigenesis; although, the underlying mechanism has not been determined [11]. Methods for autophagy detection include western blotting, biochemical assays, fluorescence microscopy, and electron microscopy [16]. STF-62247 induces cytotoxicity and reduces tumor growth of VHL-deficient RCC cells through autophagy, as detected by western blotting analysis, flow cytometry, fluorescence microscopy, and electron microscopy [12]. Here, we assessed if autophagy occurs during STF-62247-induced cell death. Conversion of the soluble form of LC3-I to its autophagic vesicle-associated form, LC3-II, is considered to be a specific marker of autophagosome formation. STF-62247 increased levels of LC3-II (lipidated LC3) in leukemia cell lines (Figure 2A). An autophagy inhibitor was added to block autophagosome–lysosome fusion and prevent lysosomal degradation of LC3, allowing for quantification of its fluorescence. Mean fluorescence intensity of LC3-II was measured using a FlowCellect Autophagy LC3 Antibody-Based Assay Kit. To facilitate monitoring of lipidated LC3-II, cells were pre-incubated for 30 min with the autophagy flux inhibitor provided in the specified kit. LC3-II accumulation was increased in the presence of STF-62247 (Figure 2B). Autophagy detection was also performed using a Cyto-ID Autophagy Detection Kit. Cyto-ID Green autophagy dye was validated by observing co-localization of the dye and RFP-LC3 in HeLa cells using fluorescence microscopy [17]. Autophagy levels also increased in the presence of STF-62247 in cells pre-treated with bafilomycin A1, a specific inhibitor of vacuolar proton ATPase (Figure 2C, 2D). Bafilomycin A1 was used to suppress acidification of the lysosome and autophagosome/lysosome. Thus, STF-62247 increased autophagosome accumulation and autophagy in leukemic cell lines (S1T, MT-2, and Jurkat).

### STF-62247 induced apoptosis in HTLV-1-infected cell lines

**Figure 3:**
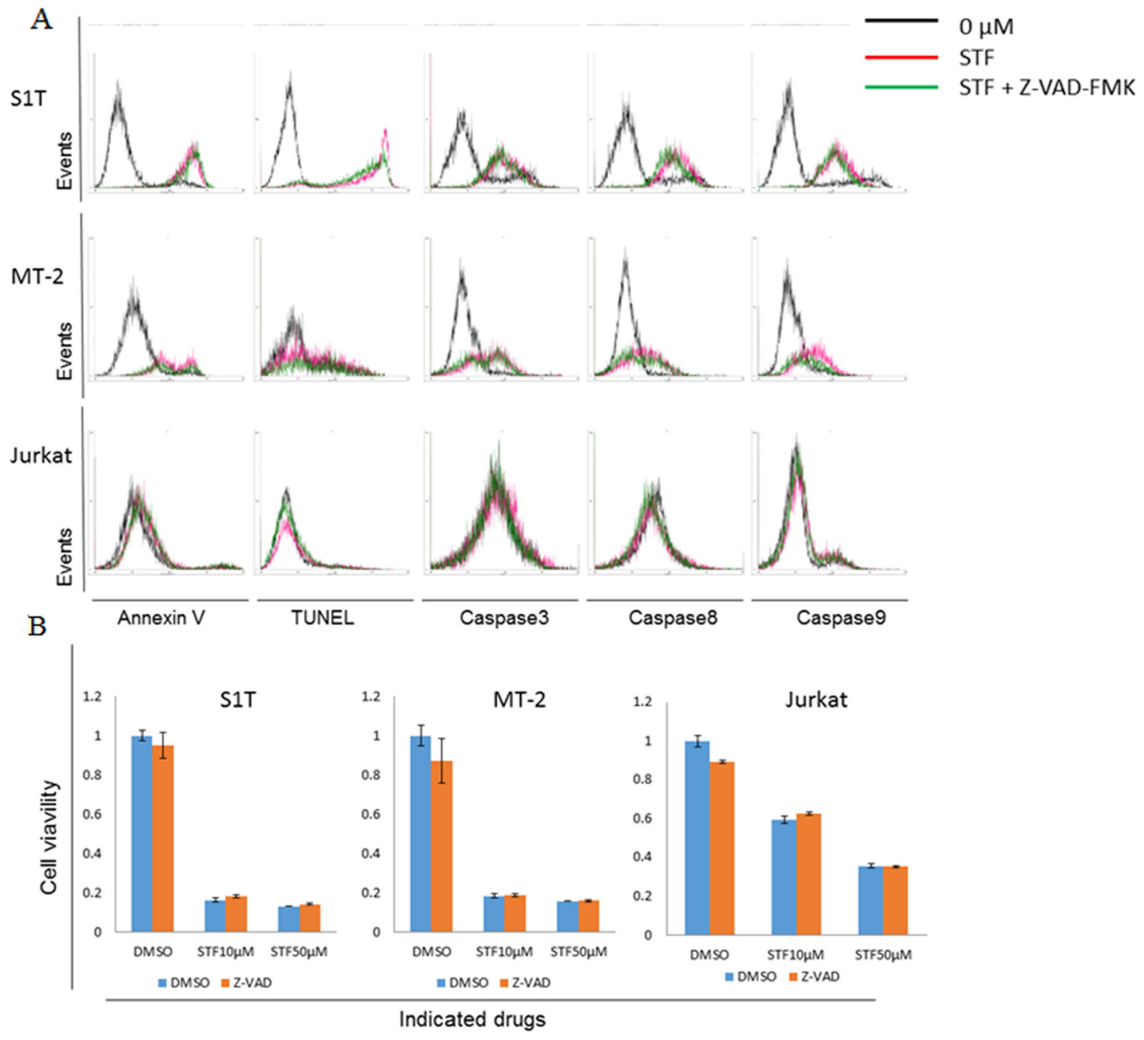
STF-62247 induced both caspase-dependent and -independent cell death **(A)** S1T, MT-2, and Jurkat cells were treated with STF-62247 (STF; S1T and Jurkat: 50 μM; MT-2: 10 μM) and Z-VAD-FMK (40 μM) for 72 h. (A) Annexin V-positive, TUNEL-positive, and caspase-positive cells were detected by flow cytometry. **(B)** Viability of cultured cells was measured by cell viability assay. Data represent mean percentage ± SD of three independent experiments.

STF-62247-induced apoptotic cell death in leukemic cell lines was analyzed by annexin V and TUNEL staining (Figure 3A). In previous studies, STF-62247 did not induce apoptosis *in vitro* in VHL-deficient cells [12]. However, we observed that STF-62247 induced phosphatidylserine externalization and DNA fragmentation in HTLV-1-infected cell lines, but not in uninfected Jurkat cells. STF-62247 also activated caspase-3, -8, and -9 in HTLV-1-infected cell lines, but not in Jurkat cells (Figure 3A).

### STF-62247 induced caspase-independent cell death

We next analyzed the effects of a pan-caspase inhibitor, Z-VAD-FMK, on STF-62247-induced cell death (Figure 3B). STF-62247 significantly inhibited cell growth and increased phosphatidylserine externalization and DNA fragmentation in HTLV-1-infected cell lines. However, the pan-caspase inhibitor did not inhibit cell death, with levels of annexin V-positive cells, DNA fragmentation, and caspase activity remaining significantly unaltered (Figure 3A). Notably, Z-VAD-FMK did suppress Fas-mediated cell death in S1T cells (data not shown). Thus, STF-62247 simultaneously induced caspase-dependent and -independent cell death mechanisms in HTLV-1-infected cell lines. These results indicate that caspase-independent cell death (CICD) may induce caspase activation; although, precise mechanisms have not yet been elucidated.

**Figure 4:**
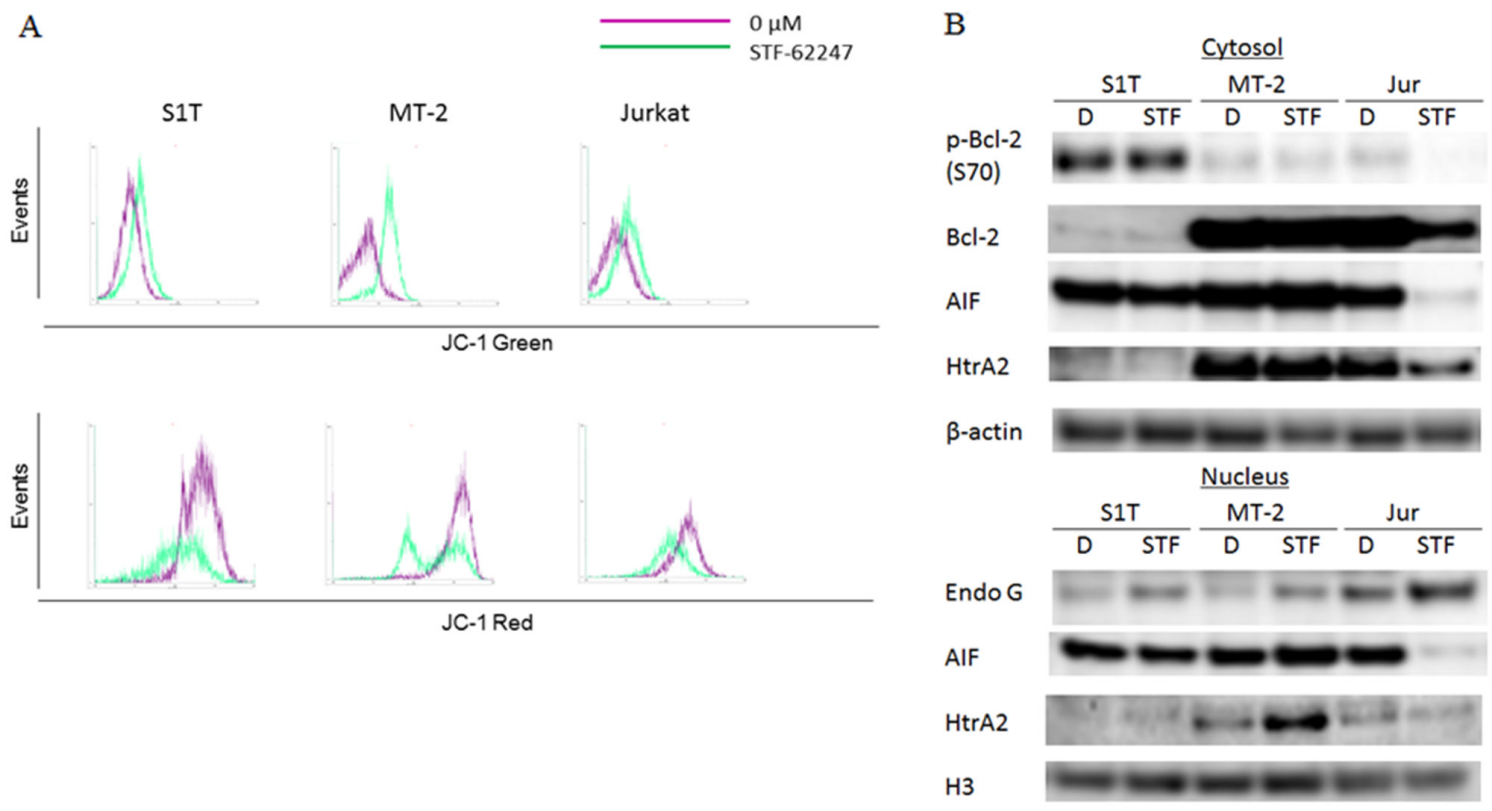
STF-62247 induced loss of mitochondrial transmembrane potential and caspase-independent cell death via endonuclease G **(A)** S1T, MT-2, and Jurkat cells were treated with STF-62247 (STF; S1T and Jurkat: 50 μM; MT-2: 10 μM) for 16 h and analyzed for JC-1 green and JC-1 red fluorescence emission components by flow cytometry. **(B)** S1T, MT-2, and Jurkat cells were treated with STF-62247 (S1T and Jurkat: 50 μM; MT-2: 10 μM) for 72 h. Protein levels were detected by western blotting with indicated antibodies.

Mitochondrial outer membrane permeabilization (MOMP) leads to the release of pro-apoptotic proteins from the mitochondrial intermembrane space, including endonuclease G, apoptosis-inducing factor (AIF) and HtrA2, which promote CICD through mechanisms that are relatively poorly defined [18]. In healthy cells with high mitochondrial transmembrane potential, JC-1 spontaneously forms complexes emitting intense red fluorescence. Conversely, in apoptotic cells with low mitochondrial transmembrane potential, JC-1 remains in its monomeric form and emits green fluorescence. By measuring the shift in fluorescence emission by flow cytometry, mitochondrial polarization was readily detected in STF-62247-treated cells (Figure 4A). The majority of STF-62247-treated cells showed a reduction in red fluorescence, indicating low mitochondrial transmembrane potential among the treated leukemia cell lines.

MOMP is controlled by the Bcl-2 family, which is composed of both pro- and anti-apoptotic proteins [19]. Notably, Bcl-2 can also inhibit autophagy [20]. STF-62247 decreased phospho-Bcl-2 (p-Bcl-2) and Bcl-2 levels in Jurkat cells, but not in S1T and MT-2 cells (Figure 4B). STF-62247 also decreased nuclear and cytosolic AIF levels in Jurkat cells, while AIF levels after STF-62247 treatment were stable in S1T and MT-2 cells. In contrast, STF-62247 increased nuclear endonuclease G levels in S1T and MT-2 cells.

**Figure 5:**
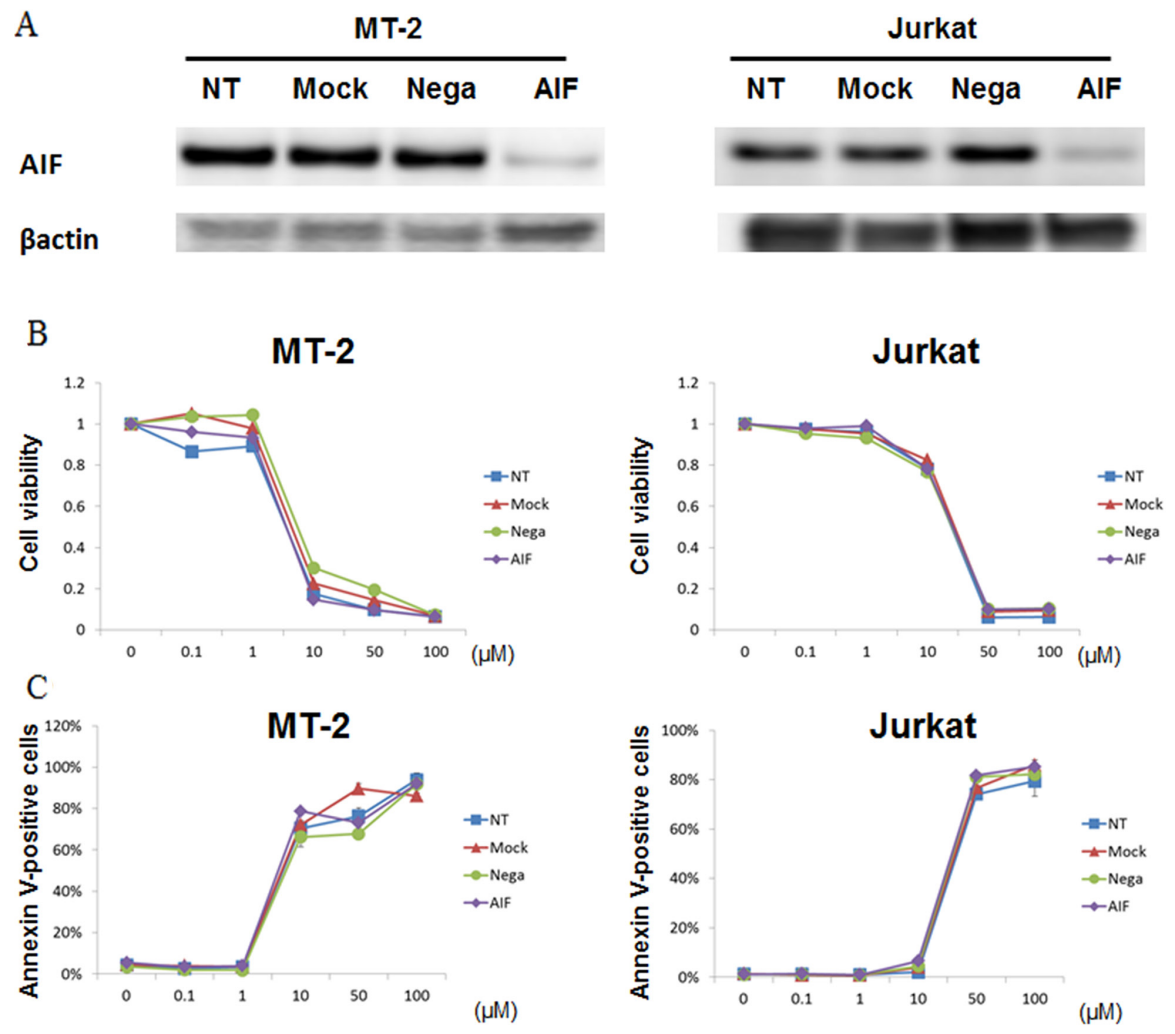
STF-62247 induced cell death in the absence of AIF protein MT-2 and Jurkat cells were either untransfected (Mock) or transfected with negative control siRNA (Nega) or siRNA against AIF. **(A)** Expression levels of AIF and β-actin were determined by western blotting. **(B)** Forty-eight hours after transfection, cells were treated with STF-62247 for 72 h. The viability of cultured cells was measured by Cell Count Reagent SF. Cells cultured in the absence of STF-62247 were assigned a relative viability of 1. **(C)** Apoptotic cells were detected by annexin V staining. Data represent mean percentage ± SD of three independent experiments.

To evaluate the relevance of AIF-mediated effects on cell death induced by STF-62247, AIF-knockdown MT-2 and Jurkat cells were treated with STF-62247. We confirmed knockdown of AIF protein in MT-2 and Jurkat cells by western blot (Figure 5A). Cell viability of MT-2 cells with non-treatment (NT), mock treatment, negative control, and AIF-knockdown 48 h after transfection was 95%, 92%, 93%, and 97%, respectively, as measured with a TC-10 automated cell counter. Cell viability of Jurkat cells with NT, mock, negative control, and AIF-knockdown 48 h after transfection was 96%, 97%, 94%, and 94%, respectively. Thus, a lack of AIF protein did not affect cell viability. Transfected cells after 48 h in each condition were treated with STF-62247 for 72 h. Cells cultured in the absence of STF-62247 under each transfection condition were assigned a relative viability of 1 (Figure 5B). STF-62247 treatment decreased the cell survival rate and increased the number of annexin V-positive cells in each condition (Figure 5C). Therefore, STF-62246–induced cell death occurred independently of AIF protein.

### STF-62247 induced cell death of primary ATL cells via apoptosis

**Figure 6:**
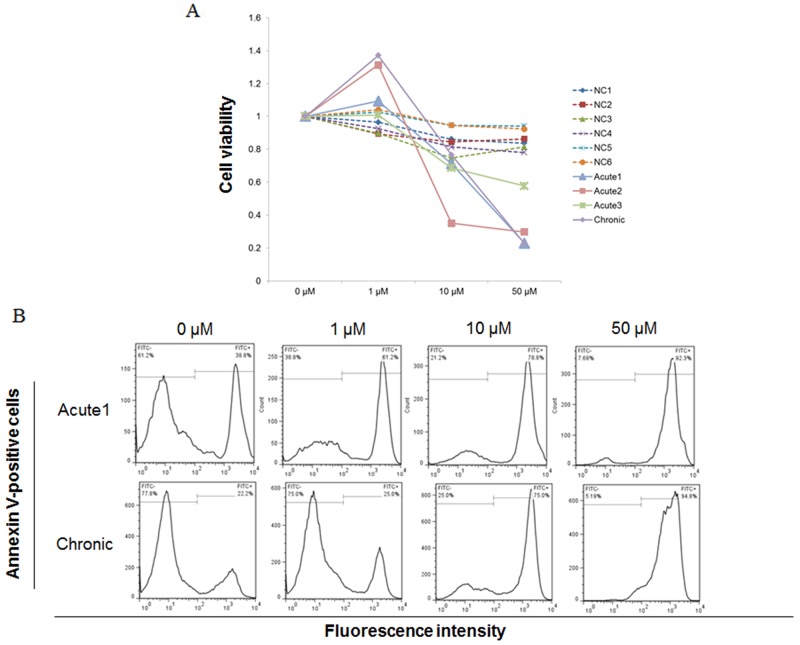
STF-62247 induced cell death in primary ATL cells Peripheral blood mononuclear cells (PBMCs) were incubated at 1 × 10^6^ cells/mL in the presence of indicated concentrations of STF-62247 for 96 h. **(A)** Viability of cultured cells was measured by Cell Count Reagent SF. Cells cultured in the absence of STF-62247 were assigned a relative viability of 1. **(B)** Annexin V-positive cells were detected in lymphocytes cultured for 72 h by flow cytometry. Data represent mean ± SD of three independent experiments.

To investigate the effect of STF-62247 on primary ATL cells, we examined whether STF-62247 affects the viability of PBMCs from acute ATL patients (Acute1−3), a chronic ATL patient (Chronic), and healthy donors (HDs). Fresh PBMCs from ATL patients were more sensitive to STF-62247 compared with control PBMCs from HDs (Figure 6A). STF-62247 showed potent activities with average GI_50_ values of 19.9, 6.9, and 21.7 μM toward PBMCs from Acute1, Acute2, and Chronic patients, respectively.

To investigate whether inhibition of cell growth occurred through enhanced apoptosis in PBMCs from ATL patients, cells treated with STF-62247 were analyzed with annexin V (Figure 6B). Representative data for Acute1 and Chronic samples are indicated in Figure 6B. The percentage of specific apoptotic cells in Acute1 after treatment with STF-62247 was 35.5% (1 μM), 64.8% (10 μM), and 87.2% (50 μM), respectively. The percentage of specific apoptotic cells in Chronic sample after treatment with STF-62247 was 31.8% (1 μM), 60.1% (10 μM) and 82.6% (50 μM), respectively. Thus, STF-62246 induced cell death by apoptosis in primary ATL cells.

## DISCUSSION

Autophagy is a tightly controlled lysosomal degradation process by which long-lived proteins, organelles, and protein aggregates are captured within autophagosomes [8, 21, 22]. While required for normal homeostasis, autophagy can contribute to the development of many pathological processes, including cancer. Alternatively, autophagy can be tumor suppressive through the elimination of oncogenic protein substrates, toxic unfolded proteins, and damaged organelles. It was previously found that STF-62247 induces cytotoxicity and reduces tumor growth of VHL-deficient RCC cells via autophagy [12]. Large numbers of autophagosomes and autolysosomes were detected in STF-62247-treated cells using electron microscopy. Here, we found that STF-62247 inhibited growth of leukemic cell lines and induced autophagy (Figures 1 and 2). The unconjugated form of ATG12 mimics BH3-only proteins and can neutralize anti-apoptotic Bcl-2 function [23]. Bcl-2 can also inhibit autophagy [20]. Notably, STF-62247 degraded Bcl-2 and p-Bcl-2 in Jurkat cells, but not in HTLV-1-infected cell lines (Figure 4B). Therefore, STF-62247-induced cell death of HTLV-1-infected cell lines occurred through Bcl-2-independent autophagy.

STF-62247 did not induce apoptosis in VHL-deficient RCC cells [12] or Jurkat cells, although it inhibited growth of HTLV-1-infected cell lines by autophagy and apoptosis (Figure 3A). Some molecules induce cell death via caspase-dependent apoptosis and CICD, such as autophagy [14, 24]. Importantly, autophagy can catabolize cellular components, such that cells eventually activate apoptotic machinery. However, a pan-caspase inhibitor did not inhibit STF-62247-induced cell death (Figure 3B). In the absence of caspase activity, apoptotic mitochondrial permeabilization is associated with enhanced autophagy through upregulation of ATG12 [25]. STF-62247-induced cell death may therefore be caspase-independent via ATG12. Furthermore, STF-62247-induced cell death was uninhibited in combination with bafilomycin A treatment for 72 hr (data not shown). Therefore, autophagy by STF-62247 can be a result, but not cause, of cell death.

Proapoptotic triggers of MOMP can engage cell death without caspase activity. While CICD shares clear similarities to apoptosis, it is distinct morphologically, biochemically, and kinetically [26]. Following MOMP, various intermembrane-space proteins are released into the cytoplasm. An active role for mitochondria has been proposed through the release of toxic proteins, such as AIF (chromatin condensation), endonuclease G (DNA degradation), and HtrA2 (loss of mitochondrial function) from the mitochondrial intermembrane space, leading to death in a caspase-independent manner [26]. STF-62247 caused disrupted Δψm (Figure 4A), and enhanced endonuclease G levels, while AIF protein levels were unchanged in S1T and MT-2 cells (Figure 4B). STF-62246 also induced cell death in the AIF-knockdown condition (Figure 5). Notably, STF-62247 enhanced the level of HtrA2 in MT-2 cells, which were more sensitive to STF-66247. These data suggest that endonuclease G is a key enzyme for STF-62247-induced DNA degradation in HTLV-1-infected cell lines.

Various therapeutic agents for ATL have been developed, including an anti-CCR4 antibody and lenalidomide [27, 28]. However, identification of new agents for ATL prevention and treatment is still required. We found that STF-62247 induced cell death via apoptosis in a caspase-independent manner, such as increased autophagy and endonuclease G levels in HTLV-1-related cell lines. Moreover, STF-62247 reduced cell viability and increased numbers of apoptotic cells in PBMCs of patients with acute ATL, which has a poor prognosis. These results suggest that STF-62247 is highly effective against HTLV-1-positive leukemic cell lines and primary ATL PBMCs. This is the first evidence to demonstrate the cell growth-inhibitory effect of an autophagy inducer by apoptosis and caspase-independent autophagic cell death in leukemic cells.

## MATERIALS AND METHODS

### Cell lines

S1T (HTLV-1-infected CD4^+^ T cell line derived from an ATL patient) [29], MT-2 (Tax producing HTLV-1-infected T cell line derived from normal human leukocytes transformed by the leukemic T cells of a patient with ATL) [30], and Jurkat (HTLV-1-uninfected T cell line) were cultured in RPMI-1640 medium supplemented with 100 units/mL penicillin, 2 mM l-glutamine, 0.1 mg/mL streptomycin, and 10% heat-inactivated fetal calf serum.

### Clinical samples

Subjects evaluated in this study included three acute-type ATL patients (median age 61 years, range 61−67, two males and one female), one chronic-type ATL patient (43 years, male), and HDs (median age 36 years, range 30−42, all males). ATL patients came to the hospital for examination of HTLV-1 infections and clinical examination, and were examined by standard serological testing for the presence of HTLV-1 and hematological analysis for diagnosis of ATL. Classification of ATL was performed according to the criteria of Shimoyama [4]. Fresh PBMCs from ATL patients were carried out in accordance with the approved guidelines of the Committees for Ethical Review of Research involving Human Subjects at Kagoshima University Hospital. All subjects provided written informed consent for participation in the project and a review of their medical records, and provided a sample of peripheral blood for isolation of PBMCs. PBMCs were obtained from peripheral blood by separation using Ficoll/Hypaque (Pharmacia, Uppsala, Sweden) density gradient centrifugation at 400 × *g* for 30 min, as previously described [31, 32]. Fresh PBMCs were used for cell viability and apoptosis analyses.

### Reagents

STF-62247, 2-[(2-Hydroxynaphthalen-1-ylmeth ylene)amino]-N-(1-phenyl-ethyl)benzamide, was purchased from Calbiochem (Merck Millipore, Darmstadt, Germany). The caspase inhibitor Z-VAD-FMK was purchased from Medical and Biological Laboratories (MBL; Nagoya, Japan), and bafilomycin A1 from Adipogen (Epalinges, Switzerland). Primary antibodies against p-Bcl-2, Bcl-2, AIF, HtrA2, endonuclease G, histone H3, and β-actin were from Cell Signaling Technology (Beverly, CA, USA), while LC-3 was from MBL. Horseradish peroxidase-conjugated anti-human antibodies were purchased from Vector Laboratories (Burlingame, CA, USA).

### Protein extraction and western blotting analysis

Whole cell lysates were obtained using RIPA Lysis Buffer (Santa Cruz Biotechnology, Dallas, TX, USA). Nuclear and cytoplasmic extracts were obtained using NE-PER Nuclear and Cytoplasmic Extraction Reagents, respectively, according to the manufacturer’s protocols (Pierce Biotechnology, Rockford, IL, USA) [24, 33]. Cell lysates were subjected to sodium dodecyl sulfate–polyacrylamide gel electrophoresis and analyzed for immunoreactivity with the appropriate primary and secondary anti-human antibodies, as indicated in the figures. Chemi-Lumi One Super (Nacalai Tesque, Kyoto, Japan) was used as a substrate.

### Cell viability assay

The effects of STF-62247 on cell viability were examined using the cell proliferation reagent Cell Count Reagent SF according to the manufacturer’s protocol (Nacalai Tesque). Absorbance at 450 nm (A_450_) was measured using an Infinite 200 PRO spectrophotometer (TECAN, Männedorf, Switzerland).

Cell viability was also measured using a TC10 automated cell counter (Bio-Rad, Hercules, CA, USA) via the trypan blue dye-exclusion method.

### Analysis of autophagy by and florescence microscopy

Autophagy was evaluated using the FlowCellect™ Autophagy LC3 Antibody-Based Assay Kit (Merck Millipore) according to the manufacturer’s instructions [34]. Briefly, discrimination between cytosolic and autophagosome-associated LC3 was achieved by monitoring translocation of LC3 using flow cytometry. As autophagy is a constitutive cellular degradation process, pretreatment of 72-h culture samples with a lysosomal inhibitor was required for 30 min prior to treatment with anti-LC3 conjugated to fluorescein isothiocyanate isomer-I (FITC) to prevent lysosomal degradation of LC3. After treatment, quantification of FITC fluorescence can then be performed. Cytosolic and autophagosomic populations are differentiated by washing cells to remove cytosolic LC3-I and retaining only membrane-bound LC3-II prior to staining.

The presence of autophagic vacuoles was assessed using a Cyto-ID Autophagy Detection Kit (Enzo Life Sciences, Farmingdale, NY, USA) according to the manufacturer’s instructions [17, 35]. Autophagy analysis was performed by incubating cells with bafilomycin A1 for 30 min at 37°C, washing cells, and analyzing fluorescence by flow cytometry using a Cell Analyzer EC800 (Sony, Tokyo, Japan) and fluorescence microscopy using a BZ-X710 fluorescence microscope (KEYENCE, Itasca, IL, USA), as previously described [14].

### Analysis of apoptosis

S1T, MT-2, and Jurkat cells (2 × 10^5^ cells) were treated with various concentrations of STF-62247. Apoptotic cells were detected by staining with annexin V-FITC (MBL), 7-amino-actinomycin D (Beckman Coulter, Fullerton, CA, USA), and a MEBSTAIN® Apoptosis TUNEL Kit Direct (MBL) and measuring fluorescence intensity with a Cell Analyzer EC800, as previously described [33]. Percentages of specific apoptotic cells were calculated as follows: % specific apoptotic cells = (annexin V-positive cells − spontaneous annexin V-positive cells) / (100 − spontaneous annexin V-positive cells) × 100.

### Detection of caspase activity

Caspase activity was evaluated using an APOPCYTO Intracellular Caspase-3, -8 Activity Detection Kit (MBL) and CaspGLOW™ Fluorescein Active Caspase-9 Staining Kit (BioVision, Milpitas, CA) according to the manufacturers’ instructions, as previously described [14].

### Mitochondrial transmembrane potential assay

Measurement of mitochondrial transmembrane potential was assessed using a JC-1 Mitochondrial Membrane Potential Assay Kit (Cayman Chemical Company, Ann Arbor, MI). Briefly, cells cultured with indicated reagents were incubated with JC-1 Staining Solution for 30 min. Apoptotic or unhealthy cells with collapsed mitochondria containing green JC-1 monomers and functional mitochondria containing red JC-1 J-aggregates were analyzed using a Cell Analyzer EC800 as previously described [14].

### Small interfering RNA (siRNA)

To silence AIF expression, a pre-designed double-stranded Human AIF Validated Stealth RNAi™ siRNA (Invitrogen, Carlsbad, CA) and Stealth RNAi™ siRNA Negative Control LO GC (Invitrogen) were employed. MT-2 or Jurkat cells were transfected with each siRNA using a Neon Transfection System (Invitrogen) as previously described [14].

### Statistical analysis

Data are expressed as mean ± SD. For data analysis, two-tailed Student’s *t* test, Mann–Whitney, and Wilcoxon matched pairs tests were performed using Excel 2007 (Microsoft Japan, Tokyo, Japan) and Statcel2 software (OMS Publishing Inc., Tokyo, Japan). In all tests, P-values of *P* < 0.05 were considered statistically significant.

## Abbreviations

AIF: apoptosis-inducing factor
ATL: adult T cell leukemia-lymphoma
CICD: caspase-independent cell death
HD: healthy donor
HTLV-1: human T cell leukemia virus
MOMP: mitochondrial outer membrane permeabilization
NT: non-treatment
PBMC: peripheral blood mononuclear cell
RCC: renal carcinoma cells
siRNA: small interfering RNA
VHL: Von Hippel-Lindau
